# Ventilator-Associated Pneumonia in Patients With Increased Intra-abdominal Pressure

**DOI:** 10.7759/cureus.81370

**Published:** 2025-03-28

**Authors:** Christos Doudakmanis, Demosthenes Makris

**Affiliations:** 1 Department of Critical Care Medicine, University Hospital of Larissa, Larissa, GRC; 2 Second Propaedeutic Department of Surgery, Laiko General Hospital of Athens, Athens, GRC

**Keywords:** intra-abdominal hypertension, intra-abdominal pressure, mechanical ventilation, multidrug resistant (mdr), ventilator-associated pneumonia

## Abstract

Increased intra-abdominal pressure (IAP) is a significant clinical concern, which has been proven to cause significant adverse events in patients. Respiratory infections are a high-yield problem in the intensive care unit (ICU). In this study, we reviewed available literature regarding the relationship between elevated IAP and the development of ventilator-associated pneumonia (VAP) in mechanically ventilated patients. Patients with prolonged mechanical ventilation are prone to develop VAP. Longer hospitalization, prior use of antibiotics, and comorbidities make these patients more susceptible to infections. Multidrug-resistant VAP poses a vast threat to critically ill patients, as it is characterized by a shift in the microbiological profile of the disease, as well as difficulties in its treatment options. Elevated IAP could adversely affect mechanically ventilated patients, as it is associated with an elevated risk of microaspirations and altered patency of the intestinal barrier, thus comprising an important factor for developing VAP. In addition, elevated IAP can deteriorate pulmonary function and hemodynamic condition of the patient, adding an extra risk for developing VAP. In such frail conditions, these patients have compromised immune function and are at risk of developing systematic infection, even resulting in the failure of multiple organs. As the microbiologic profile shifts toward multidrug-resistant bacteria, there is a need for comprehensive strategies in ICU settings to mitigate the risks associated with both elevated IAP and multidrug-resistant VAP. Timely intervention and proper management can prevent the risk of difficult-to-treat infections and life-threatening adverse events for patients.

## Introduction and background

Ventilator-associated pneumonia (VAP) is one of the most common hospital-acquired infections, with its incidence reaching as high as 40% of intubated patients. This clinical entity is associated with prolonged intensive care unit (ICU) stay and overall hospitalization and is correlated with higher morbidity and mortality. A prerequisite to differentiate VAP from any other form of hospital-acquired pneumonia is mechanical ventilation (MV) for at least 48 hours [1].

Besides the duration of MV, VAP diagnosis requires the presence of pulmonary infiltrates, clinical signs of infection, and microbiological evaluation. However, the diagnostic process remains debatable, as VAP diagnosis is highly based on clinical evaluation. The use of microbiologic confirmation remains debatable, as the endotracheal tube and respiratory tract become colonized shortly after intubation. Therefore, it is challenging to assess where a positive culture reveals the true causative pathogen versus a false-positive result following colonization [2]. Johanson et al. in their 1972 study first described VAP as nosocomial pneumonia in patients undergoing MV for at least 48 hours [3]. To update the definition to be more accurate and objective, the National Healthcare Safety Network has provided a new definition with the following specific diagnostic criteria: (a) new pulmonary infiltrates or consolidation on sequential chest X-rays; (b) fever and leukocytosis or leukopenia; and (c) new onset of purulent secretions, decompensated gas exchange, and pathological breath sounds [4]. As diagnosis is based on the evaluation of the clinical presentation, this may differ from patient to patient, adding some uncertainty. The Clinical Pulmonary Infection Score (CPIS) helps assess the probability of VAP incidence, while microbiological evaluation helps choose treatment options but has limited usefulness in the diagnostic process (Table 1) [5]. A CPIS score of 6 or more is indicative of pneumonia [6,7].

**Table 1:** Clinical Pulmonary Infection Score. WBC: white blood cells; PaO_2_/FiO_2_: arterial oxygen partial pressure/fractional inspired oxygen ratio; ARDS: acute respiratory distress syndrome This table is reproduced from Haliloglu et al. [7] published under the Creative Commons License.

Clinical parameters		Points
Temperature (°C)	36.5–38.4	0
	38.5–38.9	1
	≥39.0 and ≤36.0	2
Blood leukocytes (WBC/mm^3^)	≥4,000 and ≤11,000	0
	<4,000 or >11,000	1
Tracheal secretions	Few	0
	Moderate	1
	Large	2
	Purulent	+1
Oxygenation PaO_2_/FiO_2_	>240 or ARDS	0
	≤240 and no ARDS	2
Chest radiograph	No infiltrates	0
	Diffuse or patchy infiltrates	1
	Localized infiltrates	2
Culture of tracheal secretions	Pathogenic bacteria cultured in rare or small quantities or no growth	0
	Pathogenic bacteria cultured in moderate or large quantities	1
	Same pathogenic bacteria seen on Gram stain	2

The microbiologic basis of VAP consists of a wide spectrum of bacteria, with the majority being Gram-negative bacilli. In a significant number of VAP cases, oropharyngeal bacteria are isolated from bronchial secretion cultures. The noticeable problem, which will be augmented even more in the next few years, is the increased number of cases due to multidrug-resistant (MDR) bacteria. The incidence differs between each ICU setting and in different populations [8].

Intra-abdominal pressure (IAP) is the pressure within the abdominal cavity. This pressure is usually assessed indirectly by measuring the pressure inside the urinary bladder after instilling 25 mL of sterile saline. This measurement takes place with the patient in the supine position, at end-expiration, with no muscle contraction and with the transducer in the middle axillary line. The measurement is expressed in mmHg. In most critically ill patients, IAP is usually 5-7 mmHg. An increase in IAP values above 12 mmHg describes intra-abdominal hypertension (IAH). Further and steady increase of IAP beyond 20 mmHg is considered abdominal compartment syndrome (ACS), which can be associated with decreased abdominal perfusion pressure (APP). APP is expressed as mean arterial pressure minus IAP. Low APP values depict decreased perfusion of the organs within the abdominal cavity and can indicate subsequent organ failure or dysfunction [9].

This review explores the relationship between VAP and increased IAP, focusing on the pathophysiological mechanisms, clinical implications, and management strategies. Moreover, we investigated whether VAP caused by MDR bacteria from the intestinal flora, mainly as part of microaspirations or translocation through the intestinal barrier, present different clinical characteristics compared to VAP caused by non-MDR bacteria in patients with increased IAP.

## Review

Methodology

We conducted a comprehensive review of the literature. We conducted a literature search from 1972 up to 2024, with a focus on recently published articles in renowned medical journals. Our search was conducted using the following search engines: PubMed, Scopus Elsevier, and Cochrane Database. The key phrases for the search included “ventilator-associated pneumonia,” “multi-drug resistant bacteria,” “intra-abdominal pressure,” “intra-abdominal hypertension,” “ventilator-associated pneumonia AND intra-abdominal pressure,” “ventilator-associated pneumonia AND intra-abdominal hypertension,” “multi-drug resistant bacteria AND ventilator-associated pneumonia.” We only included articles published in the English language. We collected data derived from related clinical studies. All abstracts were reviewed by two reviewers (Christos Doudakmanis and Demosthenes Makris). Selected articles were further evaluated by the reviewers.

Ventilator-associated pneumonia: diagnosis

Given that the diagnosis of VAP remains a challenge, understanding its incidence and association with any other concerns is challenging. The National Healthcare Safety Network has provided a newer classification to create a more accurate, reliable, and applicable definition. The term ventilator-associated events (VAEs) has been proposed, which encompasses every respiratory deterioration in patients undergoing MV. VAE is subsequently stratified as ventilator-associated conditions (VACs), which is characterized by deteriorating MV parameters such as positive end-expiratory pressure and FiO_2_ and infectious ventilator-associated conditions (IVACs), which meets the criteria of VAC, in addition to signs of infection (temperature <36 or >38°C, leukocyte count <4 or >12 × 10^3^ cells/mm^3^, and addition of one or more new antibiotics for more than four days) within two days of VAC incidence. The latter is subsequently categorized into possible VAP and probable VAP. Both share some common criteria similar to those of IVAC combined with the presence of sputum or bronchoalveolar lavage with more than 25 neutrophils per field. The main differentiating criteria are that in probable VAP, there is a positive culture for specific pathogens [4].

As VAP is a hospital-acquired infection requiring endotracheal intubation for at least 48 hours, it can be further categorized based on the time of its diagnosis. Early-onset VAP is defined when diagnosed within the first five days following endotracheal intubation, whereas late-onset VAP is diagnosed after the first five days of MV [10].

In addition to the clinical evaluation of the parameters mentioned above, microbiological identification of the causative microorganism in tracheal secretion cultures is often required to confirm a VAP diagnosis. These secretions may be collected using tracheal aspiration or bronchoalveolar lavage. There is an emerging investigation of the possible utilization of biomarkers in the diagnostic process. Procalcitonin, proinflammatory cytokines, soluble triggering receptor expressed on myeloid cells type 1, and C-reactive protein are some of the biomarkers that have been studied to improve diagnostic procedures. These are non-specific biomarkers which are all increased in certain inflammatory conditions and may be altered by antibiotic administration. However, to date, no specific and ideal biomarker has been identified. The use of these biomarkers is only indicative of probable VAP, but they help monitor response to treatment. Another aspect in the diagnostic process is radiological assessment. While CT is the gold standard to diagnose VAP, chest radiograph has also increased diagnostic value. The use of lung ultrasound is a promising alternative but is highly user-dependent. Artificial intelligence (AI) and machine learning could offer significant assistance, enhancing clinical and microbiological data interpretation. Most importantly, AI could help interpret CT scans and chest radiographs to accurately diagnose new infiltrates, consolidations, or air bronchogram [11].

Ventilator-associated pneumonia: risk factors

Among the most important risk factors is the presence and prolonged duration of MV. In addition, prior use of antibiotics, prior hospitalization, elderly patients with serious comorbidities, nutritional status, surgical or trauma patients, use of central catheters and parenteral nutrition, immunosuppression, and aspiration are all potential causative mechanisms for VAP [12]. Rouzé et al. highlighted the importance of underlying pulmonary diseases such as chronic obstructive pulmonary disease in the pathogenesis of VAP. In this population, which is prone to pulmonary infections due to the colonization of their respiratory tract, the authors noticed the importance of the duration of MV, the increased incidence of microaspiration secondary to gastroesophageal reflux, as well as the impaired host defense mechanisms [13]. The patient’s body position, especially the supine position, could increase the possibility of microaspiration, and, in turn, VAP [14].

Nutritional status is a predictive factor for the course of VAP, with malnutrition being associated with adverse events. Most patients have deteriorated nutritional status, which, in turn, highlights the need for nutritional support. The main ways for this are either enteral nutrition, if feasible, or parenteral nutrition. The latter is often associated with higher rates of catheter-related infections. Malnutrition, as it is depicted by hypoalbuminemia, could also represent a frailty in a patient’s immune response. This is highly associated with disease severity [15]. Disruption of the enteral barrier due to malnutrition and systemic circulation of microorganisms with hematogenous dissemination to distant organs could be a potential mechanism [16]. However, the American Thoracic Society suggests that this is an uncommon event in patients without the presence of other contributing risk factors [8].

Ventilator-associated pneumonia: prevention

Certain interventions to prevent VAP incidence have been suggested. Elevated head bed position to avoid the supine position could assist in avoiding microaspirations of oral or gastrointestinal contents through gastroesophageal reflux. Anti-reflux prophylaxis and regular oral decontamination with chlorhexidine oral solution have also been found helpful. Critical to avoid aspiration is the use of cuffed endotracheal tubes with adequate cuff pressure. Proper hand sanitization before any contact with the patient is also important [17].

Multidrug-resistant ventilator-associated pneumonia

While VAP remains one of the most challenging complications in critically ill patients, particularly those requiring prolonged MV, the emergence of MDR pathogens has exacerbated this challenge, leading to increased morbidity, mortality, and associated healthcare costs. The altered microbiologic basis requires more potent agents to prevent antimicrobial resistance [18].

The most common pathogens causing VAP include *methicillin-resistant Staphylococcus aureus* (MRSA), *Pseudomonas aeruginosa*, *Klebsiella* species, *Escherichia* *coli*, *Acinetobacter* species, and *Enterobacter* species. Less frequently detected microorganisms include *Serratia* species, *Stenotrophomonas maltophilia, Streptococcus pneumoniae, *and *Haemophilus influenzae* [19]. The microbiologic profile shifts from each geographic region and in each ICU setting. While MRSA is very commonly detected, Gram-negative bacteria comprise the majority of VAP cases [18].

Microbiological profile and treatment strategies

Even though patients with MDR VAP may have a different microbiological profile, they share common features with non-resistant VAP cases. Fever, leukocytosis, purulent bronchial secretions, and the presence of new and progressive pulmonary infiltrates are some of the most important features. Patients are initially administered empirical antibiotic treatment, but they eventually do not respond effectively and deteriorate rapidly, worsening their treatment outcomes [20].

Gram-negative bacteria due to their unique morphologic structure are more susceptible to develop resistance. Regarding prevalence, *Klebsiella pneumoniae*, *Acinetobacter baumanii*, and *Pseudomonas aeruginosa* are the most frequently identified bacteria in tracheal secretion cultures [20]. Despite its heterogeneity, globally, the prevalence of *Pseudomonas aeruginosa* is higher compared to other Gram-negative bacteria. Infections caused by this bacterium are often resistant to multiple classes of antibiotics, making treatment a challenging endeavor [21].

Initial empirical antibiotic therapy usually includes a combination of two different classes of antibiotics, with both Gram-positive and Gram-negative pathogen coverage [22]. The resistance pattern shows strains resistant to beta-lactams and aminoglycosides and highly resistant to quinolones [20]. Rao et al. presented long-term data on MDR strains. While their findings are in accordance with previous studies, they highlighted up to 100% resistance to aminoglycosides and quinolones and the increasing resistance to ampicillin and third-generation cephalosporines. On the other hand, no signs of resistance were detected for antibiotics specific for MRSA, such as vancomycin, teicoplanin, and linezolid [18].

Antibiotic therapy for cases of MDR VAP is based on available microbiological data of each ICU setting and is later modified based on the culture results in an individualized manner. For Gram-positive bacteria causing VAP, vancomycin or linezolid should be administered. In cases of VAP caused by Gram-negative bacteria, treatment modalities highly depend on microbiologic data and the bacteria’s susceptibility to antibiotics. In these cases, carbapenems are highly suggested. In non-responders or septic shock cases, a combination of two or more antibiotics is preferred. The duration of the treatment should be at least seven days, following which de-escalation should be considered [22,23].

Risk factors for developing multidrug-resistant ventilator-associated pneumonia

In terms of demographic parameters and coexisting comorbidities, MDR VAP affect patients equally in comparison with non-resistant VAP [24,25]. It is well supported in the literature that prior use of antibiotics is an independent risk factor for developing MDR VAP; however, Depuydt et al. and Grgurich et al. define it as the cornerstone to the process [26-28]. While VAP is reported as a complication in around 30% of ICU patients according to several studies, a significant 44% of the cases are attributed to MDR pathogens [26]. In addition to prior use of antibiotics, incorrect use of empirical antibiotics is considered a risk factor for developing MDR VAP [29].

Prior lower respiratory tract infection and acute respiratory distress syndrome are risk factors for developing MDR VAP [22]. The locoregional circumstances, i.e., the hospital environment and ICU settings, are also characterized by specific MDR pathogens, derived from the use of specific antibiotics in these settings. Use of quinolones and carbapenems is also a stratified risk factor. On the other hand, prolonged hospitalization and ICU stay could also play a significant role [30]. In addition, administration of corticosteroids is associated with increased incidence of MDR VAP [29] (Tables 2, 3).

**Table 2:** Risk factors for MDR VAP. MDR: multidrug resistant; VAP: ventilator-associated pneumonia; ARDS: acute respiratory distress syndrome Table credits: Christos Doudakmanis

Summary of the most common risk factors
Prolonged length of hospitalization
Prior use of antibiotics
Use of corticosteroids
Renal replacement for acute renal failure before VAP diagnosis
Sepsis and septic shock at the time of VAP diagnosis
ARDS preceding VAP

**Table 3:** Characteristics of patients with MDR VAP in comparison with patients with non-MDR VAP. MDR: multi-drug resistant; VAP: ventilator-associated pneumonia; MV: mechanical ventilation This table has been reproduced from Moreira and Gontijo Filho [29] published under the Creative Commons License.

Clinical characteristics and outcomes of patients with MDR VAP
Equal incidence in both genders
Longer hospital stay
Tendency for higher MV duration
More frequent prior use of antibiotics
More frequent use of corticosteroids
Most cases had inappropriate initial antibiotic therapy
Tendency for late-onset MDR VAP
Higher mortality

Impact of multidrug-resistant ventilator-associated pneumonia

MDR VAP could further be characterized as monobacterial or polybacterial. Patients with polybacterial MDR VAP are characterized by higher mortality compared to those with monobacterial MDR VAP. Another interesting feature is that this mortality rate is increased in shorter time intervals due to disease severity. The overall worse outcomes of patients with polybacterial MDR VAP highlight the possible interaction of multiple pathogens, which is attributed to the altered efficacy of antibiotics due to the metabolites produced by the pathogens, possible synergy, biofilm production, and altered intrinsic signals in the pathway of immune response [31].

Another important factor that should be highlighted is the severity of the patient’s illness. Patients with Acute Physiology and Chronic Health Evaluation II (APACHE-II) score 17 or higher on day three of their ICU stay are categorized as being at high risk of fatality. APACHE-II scores of over 16 in patients with VAP are an independent predictor of mortality [32]. The Charlson Comorbidity Index (CCI) indicates a more severe condition and eventually a worse prognosis. A CCI score of above 5 is considered an indicative cut-off point for worse outcomes [33]. The combination of two or more antibiotics in patients, in addition to previously administered regimens, could lead to a higher incidence of MDR VAP. Critically ill patients often present with acute kidney injury, which in many cases requires renal replacement therapy, thus posing a deteriorating factor for MDR VAP development [21]. Furthermore, the longer the patient is on MV, the higher the relative risk of infection by MDR organisms [34]. In the ICU setting, other types of infections potentially caused by MDR bacteria, such as urinary tract infections and catheter-related bloodstream infections, may coexist and facilitate VAP [35]. Bloodstream infections and septic shock caused by these microorganisms are also a potential causative factor for developing VAP [22].

Sepsis is a life-threatening condition which can lead to multiorgan failure. Patients with sepsis are prone to develop VAP. Early-onset VAP depicts the severity of sepsis, as it is related to worse outcomes in terms of mortality, when patients with late-onset VAP are affected by prolonged MV and ICU stay [36]. Raghuram et al. reported that the incidence of sepsis in patients with MDR VAP was up to 67%, and their combination led to higher rates of multiorgan failure and overall mortality [37]. On the other hand, up to 51% of patients with VAP were at risk of developing severe sepsis and septic shock. CPIS depicts with relative accuracy the severity, with increased values associated with a higher risk of septic shock. Sepsis and septic shock alter the treatment regimens. In patients at low risk of MDR VAP, treatment regimens include empiric monotherapy with carbapenems or fluoroquinolones, which is modified to broad-spectrum antibiotics targeting Gram-negative pathogens in combination with MRSA-targeting drugs in higher risk patients. In case of septic shock, dual broad-spectrum antibiotics for Gram-negative bacteria should be administered combined with antibiotics targeting Gram-positive bacteria. A potential approach to prevent the development of MDR infections and subsequently MDR VAP, especially in patients with long-term hospitalization, such as patients with elevated IAP, could be a “hit hard and strong” approach. This could offer fast control of the infections, thereby potentially avoiding long treatment duration or recurrence. However, we acknowledge that this strategy needs further support in future studies [38].

Pathophysiology and potential association between elevated intra-abdominal pressure and ventilator-associated pneumonia

IAP is a significant concern in critically ill patients, and its interplay with VAP presents complex clinical challenges. IAP is defined as the measured pressure within the abdominal cavity [39]. Both direct and indirect methods of IAP have been proposed. While direct methods have increased accuracy, they are far more invasive and costly. Indirect measurement using the urinary bladder pressure measurement as a reference point, as proposed by Kron et al., is recommended. This technique is easily reproducible and cost-effective, but it is highly dependent on urinary bladder compliance and the patient’s position. IAP values are measured using pressure transducers connected to the urinary catheter or using fluid columns. Measurements usually occur intermittently [40]. However, continuous IAP measurement has also been studied, with similar results [41].

Under normal conditions, IAP is well under 12 cmH_2_O. Elevated IAP, often defined as IAH when it exceeds 12 mmHg, can lead to ACS, when pressures exceed 20 mmHg, causing significant organ dysfunction. Papakrivou et al. assessed increased IAP as an independent risk factor for VAP through translocation of bacteria from the intestinal flora into the bloodstream. Elevated IAP can disrupt the intestinal barrier, promoting bacterial translocation and systemic infection, which can complicate infection control. Patients with VAP and elevated IAP tend to have a different microbiologic profile compared to those with normal IAP, and this profile usually comprises bacteria from the intestinal flora. On the other hand, there is a shift in the microbiologic profile in patients with normal IAP, as causative microorganisms are usually not from the intestinal flora [39].

Oropharyngeal and upper gastrointestinal content aspiration has been implicated in VAP pathogenesis through transcolonization to the respiratory tract [42]. In our previously published study, we explored the correlation between increased IAP and VAP. Increased IAP can impair gastrointestinal motility, leading to ileus and increased risk of aspiration of gastric and intestinal contents, further complicating respiratory function. Increased IAP, even within normal limits, can cause VAP through microaspirations. This study also provided data supporting that patients who developed VAP had higher mean IAP values. This correlation is even more significant, taking into consideration that 53% of patients with VAP developed IAH during their ICU stay. Length of stay is an indicator of a complicated ICU course. VAP results in significantly more days in the ICU, with these patients remaining under MV for a longer duration [43]. In a study from the same center, Papakrivou et al. reported that the incidence of VAP in their study population was 36.6%, while in patients with elevated IAP, it increased to 53.3%. At the same time, another finding of this study was that 19.5% of patients had VAP and increased IAP. To put this into perspective, this incidence rate is high, considering that these data came from a center with a high frequency of infections caused by MDR bacteria, especially *Klebsiella* species. In the same study, in 54% of the cases with both VAP and increased IAP, MDR *Klebsiella* species were isolated, while these bacteria were found only in 9.5% of patients with VAP but with normal IAP [39]. Following personal communication, we reviewed data used in a previous study in our center. Of the total 20 patients who developed VAP, in 35% (7/20), VAP was caused by MDR bacteria. Mean IAP in patients with MDR VAP was 10.14 ± 3.89 mmHg, while mean IAP was 5.30 ± 3.27 mmHg in non-MDR VAP. This was associated with a statistically significant difference (p = 0.002), meaning that in the study sample, patients with elevated IAP tended to develop MDR VAP more frequently. Notably, three of the seven MDR VAP patients developed IAH, while all patients with non-MDR VAP had normal IAP measurements [43].

Abdominal compliance is a measure of susceptibility of the abdominal cavity to distention in response to fluctuations of abdominal volume per change in IAP, and it is highly dependent on the abdominal wall and the diaphragm [44]. Elevated IAP exerts pressure on the diaphragm, reducing intrathoracic volume and lung compliance. In addition, it can lead to atelectasis and impaired gas exchange, exacerbating hypoxemia and hypercapnia in patients with VAP. As a result, there is difficulty in ventilating the patient effectively. The elevated pressure on the diaphragm, along with the reduced volume of the thoracic cavity, can also alter hemodynamic parameters. Due to the diminished intrathoracic volume, the intrathoracic pressure increases. As a result, venous return to the heart is reduced and leads to decreased cardiac output, causing hypotension and lowered perfusion of the vital organs. This impaired perfusion can lead to multiorgan dysfunction, further deteriorating the status of the critically ill patient [45]. Decreased renal perfusion should be highlighted. Increased IAP can reduce renal blood flow, leading to acute kidney injury (AKI). This complicates the management of fluid balance and the administration of nephrotoxic antibiotics often required to treat VAP [46,47].

**Figure 1:**
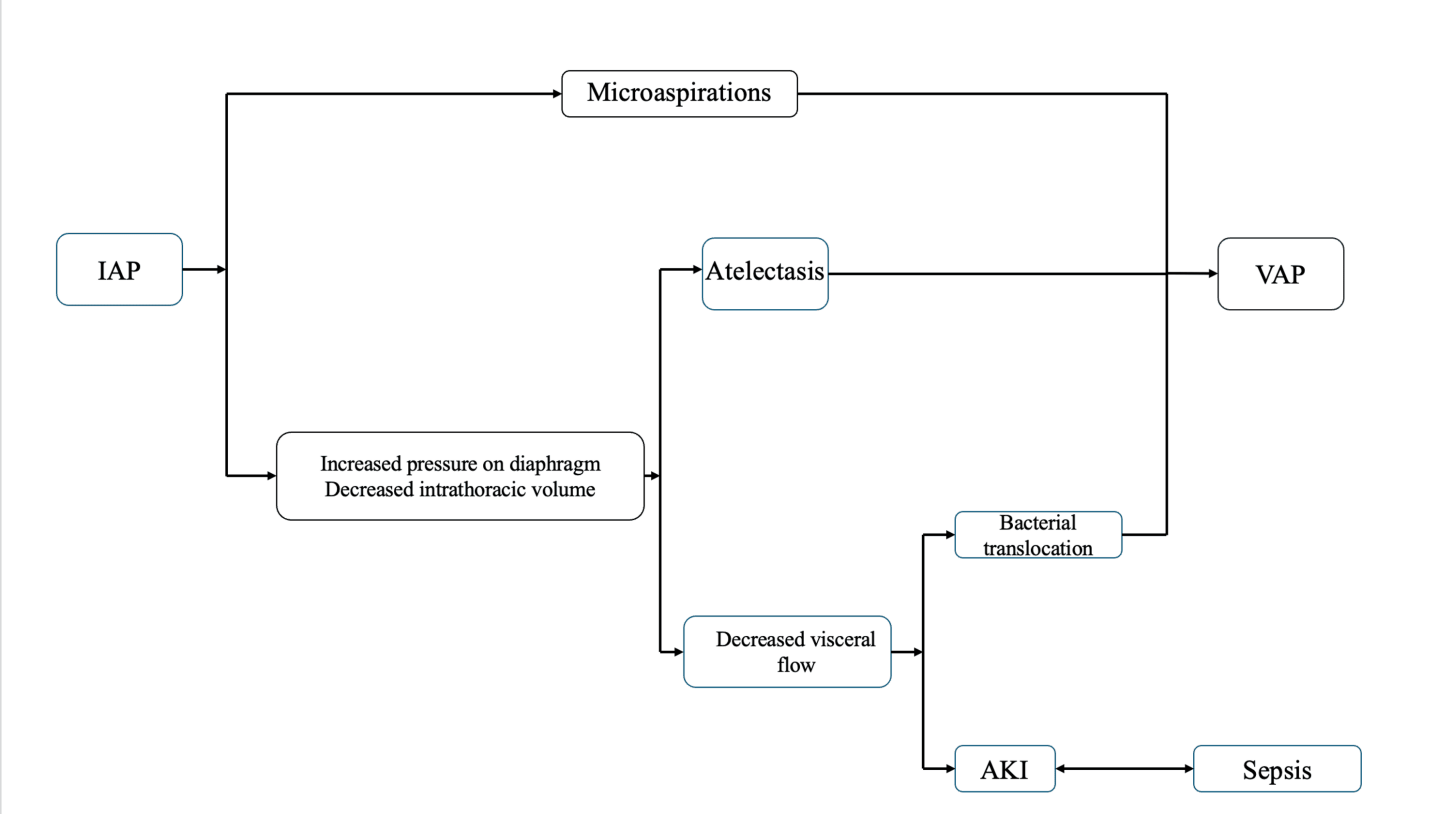
Pathophysiology and association between elevated IAP and VAP. IAP: intra-abdominal pressure; VAP: ventilator-associated pneumonia; AKI: acute kidney injury Figure credits: Christos Doudakmanis

Gursel et al. reported that AKI is diagnosed in 38% of VAP patients. In the study, MDR VAP and sepsis were correlated with a higher incidence of AKI and were stratified as independent risk factors [46]. On the other hand, Younan et al. proposed that the incidence of AKI is a predictor of VAP severity [48]. Due to the relationship between sepsis, VAP, and AKI, there is a need to monitor biochemical parameters, which will be first affected in the course of the disease, so that preventive actions and quick interventions can be initiated even before the urea creatinine rises. Renal function and AKI can be assessed by evaluating urine output and serum creatinine values. A decline in urine output may precede the onset of AKI for at least 12 hours and can be the first sign of affected renal function. On the other hand, serum creatinine is usually increased gradually [49]. Elevated serum creatinine levels are observed when there is at least a 50% loss of renal function. As sepsis reduces the production rate of creatinine, even a slight increase in serum creatinine levels indicates a poor prognosis. Other useful biomarkers include blood urea nitrogen and serum lactate, which have relatively good sensitivity and indicate disease severity [50] (Figure 1).

Important factors associated with multidrug-resistant bacteria

Patients with IAP measurements above normal limits usually have impaired immune function, and dysfunction of the intestinal barrier promotes the translocation of intestinal bacteria into the bloodstream, leading to systemic infection [51]. Bacteria passing from the intestine to the bloodstream can cause VAP, and, in many cases, these strains show resistance to certain antibiotics. This resistance profile is explained either by previously administered antibiotic regimens or by specific drug-resistant gene phenotypes [30].

As patients in ICUs are becoming colonized with MDR bacteria, intestinal colonization is also of concern. Intestinal colonization with MDR bacteria is the cornerstone of both intestinal and non-intestinal infections. If the patient’s intestine is colonized, infections are most predominantly caused by MDR bacteria, rather than by other non-resistant strains. Increased IAP, especially IAH, are associated with alterations in the visceral blood flow, as it can provoke intestinal ischemia through reduced mesenteric vasculature and portal vein flow. Increases in IAP could demonstrate a risk for bacterial translocation and gut-induced lung injury. These mechanisms affect patients undergoing surgical interventions and operations, and, most importantly, laparotomy [52]. 

The increased VAP incidence is a consequence of translocation of bacteria through the bloodstream and aspirations of oropharyngeal and gastrointestinal contents in the presence of elevated IAP. Patients with elevated IAP or IAH are often critically ill and have multiple comorbidities. Furthermore, these patients often require longer periods under MV and overall duration of hospitalization. Elevated IAP levels are associated with a higher incidence of VAP. Patients with VAP also have higher mortality rates. Patients with increased IAP share the same feature. Of note, the subgroup of patients who have both VAP and elevated IAP is also characterized by increased mortality [39,43]. We speculate that patients with increased IAP share common characteristics with long-hospitalized ICU patients; a potential infection, such as VAP, could be, unfortunately, an MDR infection. The physiological changes associated with increased IAP can affect the pharmacokinetics and pharmacodynamics of antibiotics, making it difficult to achieve effective drug concentrations at the infection site. Increased IAP and IAH may cause decreased APP. A drop in APP can decrease visceral blood flow and perfusion and thus decrease drug bioavailability to abdominal organs. Secondary to this, fluctuations in the visceral blood flow, which can cause organ dysfunction, may raise the need to adjust the dosages of certain antibiotics to avoid toxicity. The interconnection of increased IAP and MDR VAP can lead to prolonged ICU stay, which at the same time predisposes to colonization by MDR bacteria and a higher risk for MDR infection. The combined burden is associated with higher treatment costs and worse outcomes [20,44,53]. Despite the established link between elevated IAP and VAP, there are only a few studies on the topic. We acknowledge that this poses a limitation of our study. To our knowledge, there is no established correlation between VAE and increased IAP, and we hope that our review forms the basis for future research.

## Conclusions

Critically ill patients with increased IAP and MDR VAP are considered a challenging patient population in every ICU setting. Patients with MDR VAP tend to present differently in comparison with non-MDR VAP patients. Most cases are late-onset VAP, with prior use of antibiotics. In many patients, the initial therapy was not optimal. These patients stay longer in the hospital and present higher mortality rates. Understanding the pathophysiological interplay between increased IAP and VAP is essential for effective management. After reviewing data following personal communication with authors from a previous study, there is an initial insight that there is a potential positive correlation between elevated IAP and incidence of MDR VAP. Increased IAP and IAH could cause VAP through translocation and gastroesophageal reflux. Translocation is the result of reduced APP, decreased visceral blood flow, and disruption of the gastrointestinal barrier. While elevated IAP and IAH could cause organ dysfunction, they could also alter the bioavailability of antibiotics in target organs. By employing targeted strategies to monitor and reduce IAP, optimize ventilation, provide hemodynamic support, and adhere to antibiotic stewardship and infection control measures, healthcare providers can improve outcomes for these vulnerable patients. To our knowledge, there are no published data on this topic. Hence, targeted large-scale studies should explore this correlation. With the emergence of MDR bacteria, continued research and innovation are needed to develop more effective interventions and improve survival rates in this high-risk group.

## References

[1] Papazian L, Klompas M, Luyt CE (2020). Ventilator-associated pneumonia in adults: a narrative review. Intensive Care Med.

[2] Jorens PG (2016). Sticking to an old definition of ventilator-associated pneumonia is not old-fashioned. Respir Care.

[3] Johanson WG Jr, Pierce AK, Sanford JP, Thomas GD (1972). Nosocomial respiratory infections with gram-negative bacilli. The significance of colonization of the respiratory tract. Ann Intern Med.

[4] Spalding MC, Cripps MW, Minshall CT (2017). Ventilator-associated pneumonia: new definitions. Crit Care Clin.

[5] Fernando SM, Tran A, Cheng W (2020). Diagnosis of ventilator-associated pneumonia in critically ill adult patients-a systematic review and meta-analysis. Intensive Care Med.

[6] Nair GB, Niederman MS (2015). Ventilator-associated pneumonia: present understanding and ongoing debates. Intensive Care Med.

[7] Haliloglu M, Bilgili B, Bilginer H (2020). A new scoring system for early diagnosis of ventilator-associated pneumonia: LUPPIS. Arch Med Sci.

[8] Wu H, Harder C, Culley C (2017). The 2016 clinical practice guidelines for management of hospital-acquired and ventilator-associated pneumonia. Can J Hosp Pharm.

[9] Kirkpatrick AW, Roberts DJ, De Waele J (2013). Intra-abdominal hypertension and the abdominal compartment syndrome: updated consensus definitions and clinical practice guidelines from the World Society of the Abdominal Compartment Syndrome. Intensive Care Med.

[10] Gunalan A, Sastry AS, Ramanathan V, Sistla S (2023). Early- vs late-onset ventilator-associated pneumonia in critically ill adults: comparison of risk factors, outcome, and microbial profile. Indian J Crit Care Med.

[11] Howroyd F, Chacko C, MacDuff A (2024). Ventilator-associated pneumonia: pathobiological heterogeneity and diagnostic challenges. Nat Commun.

[12] Vincent JL, Lobo S, Struelens M (2001). Ventilator associated pneumonia: risk factors and preventive measures. J Chemother.

[13] Rouzé A, Cottereau A, Nseir S (2014). Chronic obstructive pulmonary disease and the risk for ventilator-associated pneumonia. Curr Opin Crit Care.

[14] Torres A, Serra-Batlles J, Ros E (1992). Pulmonary aspiration of gastric contents in patients receiving mechanical ventilation: the effect of body position. Ann Intern Med.

[15] Ścisło L, Walewska E, Bodys-Cupak I, Gniadek A, Kózka M (2022). Nutritional status disorders and selected risk factors of ventilator-associated pneumonia (VAP) in patients treated in the intensive care ward-a retrospective study. Int J Environ Res Public Health.

[16] Nagpal R, Yadav H (2017). Bacterial translocation from the gut to the distant organs: an overview. Ann Nutr Metab.

[17] Osman S, Al Talhi YM, AlDabbagh M, Baksh M, Osman M, Azzam M (2020). The incidence of ventilator-associated pneumonia (VAP) in a tertiary-care center: comparison between pre- and post-VAP prevention bundle. J Infect Public Health.

[18] Rao SV, Thilakchand KR, Boloor R (2024). Antimicrobial resistance pattern in aerobic bacteria isolated from endotracheal aspirate in ventilator-associated pneumonia: ten years observation from a tertiary care hospital. J Anaesthesiol Clin Pharmacol.

[19] Jones RN (2010). Microbial etiologies of hospital-acquired bacterial pneumonia and ventilator-associated bacterial pneumonia. Clin Infect Dis.

[20] Vo TP, Dinh TC, Phan HV, Cao TT, Duong PT, Nguyen T (2022). Ventilator-associated pneumonia caused by multidrug-resistant gram-negative bacteria in Vietnam: antibiotic resistance, treatment outcomes, and colistin-associated adverse effects. Healthcare (Basel).

[21] Li Y, Roberts JA, Walker MM, Aslan AT, Harris PN, Sime FB (2024). The global epidemiology of ventilator-associated pneumonia caused by multi-drug resistant Pseudomonas aeruginosa: a systematic review and meta-analysis. Int J Infect Dis.

[22] Kalil AC, Metersky ML, Klompas M (2016). Management of adults with hospital-acquired and ventilator-associated pneumonia: 2016 Clinical Practice Guidelines by the Infectious Diseases Society of America and the American Thoracic Society. Clin Infect Dis.

[23] Garnacho-Montero J, Corcia-Palomo Y, Amaya-Villar R, Martin-Villen L (2014). How to treat VAP due to MDR pathogens in ICU patients. BMC Infect Dis.

[24] Kajeekul R, Thamlikitkul V, Wonglaksanapimon S, Rattanaumpawan P (2024). Epidemiology of ventilator-associated tracheobronchitis and ventilator-associated pneumonia caused by multidrug-resistant Gram-negative bacteria at a tertiary care hospital in Thailand. JAC Antimicrob Resist.

[25] Breijyeh Z, Jubeh B, Karaman R (2020). Resistance of Gram-negative bacteria to current antibacterial agents and approaches to resolve it. Molecules.

[26] Patil HV, Patil VC (2017). Incidence, bacteriology, and clinical outcome of ventilator-associated pneumonia at tertiary care hospital. J Nat Sci Biol Med.

[27] Depuydt PO, Vandijck DM, Bekaert MA, Decruyenaere JM, Blot SI, Vogelaers DP, Benoit DD (2008). Determinants and impact of multidrug antibiotic resistance in pathogens causing ventilator-associated-pneumonia. Crit Care.

[28] Grgurich PE, Hudcova J, Lei Y, Sarwar A, Craven DE (2012). Management and prevention of ventilator-associated pneumonia caused by multidrug-resistant pathogens. Expert Rev Respir Med.

[29] Moreira MR, Gontijo Filho PP (2012). Multidrug-resistant pathogens causing ventilator-associated pneumonia: risk factors, empirical antimicrobial therapy and outcome of patients in an intensive care unit (ICU) of Brazilian university hospital. Int J Med Med Sci.

[30] Hu JN, Hu SQ, Li ZL, Bao C, Liu Q, Liu C, Xu SY (2023). Risk factors of multidrug-resistant bacteria infection in patients with ventilator-associated pneumonia: a systematic review and meta-analysis. J Infect Chemother.

[31] Adukauskiene D, Ciginskiene A, Adukauskaite A, Koulenti D, Rello J (2023). Clinical features and outcomes of VAP due to multidrug-resistant Klebsiella spp.: a retrospective study comparing monobacterial and polybacterial episodes. Antibiotics (Basel).

[32] Gursel G, Demirtas S (2006). Value of APACHE II, SOFA and CPIS scores in predicting prognosis in patients with ventilator-associated pneumonia. Respiration.

[33] Núñez SA, Roveda G, Zárate MS, Emmerich M, Verón MT (2021). Ventilator-associated pneumonia in patients on prolonged mechanical ventilation: description, risk factors for mortality, and performance of the SOFA score. J Bras Pneumol.

[34] Assefa M (2022). Multi-drug resistant gram-negative bacterial pneumonia: etiology, risk factors, and drug resistance patterns. Pneumonia (Nathan).

[35] Suryawanshi VR, Pawar A, Purandare B, Vijayvargiya N, Sancheti S, Philip S, Nagare P (2024). Microbial profile, antimicrobial susceptibility, and prevalence of MDR/XDR pathogens causing medical device associated infections: a single center study. Indian J Crit Care Med.

[36] Fang WF, Fang YT, Huang CH (2020). Risk factors and associated outcomes of ventilator-associated events developed in 28 days among sepsis patients admitted to intensive care unit. Sci Rep.

[37] Aydogdu M, Gursel G (2008). Predictive factors for septic shock in patients with ventilator-associated pneumonia. South Med J.

[38] Raghuram A, Gnoni M, Wiemken TL (2017). Sepsis in patients with ventilator associated pneumonia due to methicillin-resistant Staphylococcus aureus: incidence and impact on clinical outcomes. Univ Louisville J Resp Infect.

[39] Papakrivou E, Makris D, Manoulakas E, Karvouniaris M, Zakynthinos E (2020). Intra-abdominal hypertension is a risk factor for increased VAP incidence: a prospective cohort study in the ICU of a tertiary hospital. J Intensive Care Med.

[40] Li Z, Wang H, Lu F (2024). The development, feasibility and credibility of intra-abdominal pressure measurement techniques: a scoping review. PLoS One.

[41] Balogh Z, Jones F, D'Amours S, Parr M, Sugrue M (2004). Continuous intra-abdominal pressure measurement technique. Am J Surg.

[42] Estes RJ, Meduri GU (1995). The pathogenesis of ventilator-associated pneumonia: I. Mechanisms of bacterial transcolonization and airway inoculation. Intensive Care Med.

[43] Doudakmanis C, Stamatiou R, Makri A (2023). Relationship between intra-abdominal pressure and microaspiration of gastric contents in critically ill mechanically ventilated patients. J Crit Care.

[44] Blaser AR, Björck M, De Keulenaer B, Regli A (2015). Abdominal compliance: a bench-to-bedside review. J Trauma Acute Care Surg.

[45] Łagosz P, Sokolski M, Biegus J, Tycinska A, Zymlinski R (2022). Elevated intra-abdominal pressure: a review of current knowledge. World J Clin Cases.

[46] Gursel G, Demir N (2006). Incidence and risk factors for the development of acute renal failure in patients with ventilator-associated pneumonia. Nephrology (Carlton).

[47] Zhang J, Song L, Ma Z (2024). Intra-abdominal pressure and residual renal function decline in peritoneal dialysis: a threshold-based investigation. Ren Fail.

[48] Younan D, Delozier SJ, Adamski J (2020). Factors predictive of ventilator-associated pneumonia in critically ill trauma patients. World J Surg.

[49] Wang J, Niu D, Li X (2024). Effects of 24-hour urine-output trajectories on the risk of acute kidney injury in critically ill patients with cirrhosis: a retrospective cohort analysis. Ren Fail.

[50] Li J, Zhu M, Yan L (2024). Predictive models of sepsis-associated acute kidney injury based on machine learning: a scoping review. Ren Fail.

[51] Li H, Chen Y, Huo F, Wang Y, Zhang D (2017). Association between acute gastrointestinal injury and biomarkers of intestinal barrier function in critically ill patients. BMC Gastroenterol.

[52] Zheng R, Jiang Y, Yan C, Li Y, Song X, Zheng P (2023). Intra-abdominal hypertension contributes to the development of ventilator-associated pneumonia from intestinal bacteria. Infect Drug Resist.

[53] Bezerra IL, Nassar Junior AP, Dos Santos TM (2024). Patient-level cost analysis of intensive care unit acquired infections: a prospective cohort study [in press]. J Hosp Infect.

